# ASSET (Age/Sex Standardised Estimates of Treatment): A Research Model to Improve the Governance of Prescribing Funds in Italy

**DOI:** 10.1371/journal.pone.0000592

**Published:** 2007-07-04

**Authors:** Giampiero Favato, Paolo Mariani, Roger W. Mills, Alessandro Capone, Matteo Pelagatti, Vasco Pieri, Alberico Marcobelli, Maria G. Trotta, Alberto Zucchi, Alberico L. Catapano

**Affiliations:** 1 School of Projects, Processes and Systems, Henley Management College, Henley-on-Thames, United Kindgom; 2 Department of Statistical Science, Bicocca University, Milan, Italy; 3 Servizio di Epidemiologia e Farmacia Preventiva (SEFAP), Milan, Italy; 4 Regional Health Authority (ASSR) Marche, Ancona, Italy; 5 Regional Health Authority (ASSR) Basilicata, Potenza, Italy; 6 Local Health Authority (ASL) Milano 3, Monza, Italy; 7 Department of Pharmacological Sciences, University of Milan, Milan, Italy; Norwegian Knowledge Centre for the Health Services, Norway

## Abstract

**Background:**

The primary objective of this study was to make the first step in the modelling of pharmaceutical demand in Italy, by deriving a weighted capitation model to account for demographic differences among general practices. The experimental model was called ASSET (Age/Sex Standardised Estimates of Treatment).

**Methods and Major Findings:**

Individual prescription costs and demographic data referred to 3,175,691 Italian subjects and were collected directly from three Regional Health Authorities over the 12-month period between October 2004 and September 2005. The mean annual prescription cost per individual was similar for males (196.13 euro) and females (195.12 euro). After 65 years of age, the mean prescribing costs for males were significantly higher than females. On average, costs for a 75-year-old subject would be 12 times the costs for a 25–34 year-old subject if male, 8 times if female. Subjects over 65 years of age (22% of total population) accounted for 56% of total prescribing costs. The weightings explained approximately 90% of the evolution of total prescribing costs, in spite of the pricing and reimbursement turbulences affecting Italy in the 2000–2005 period. The ASSET weightings were able to explain only about 25% of the variation in prescribing costs among individuals.

**Conclusions:**

If mainly idiosyncratic prescribing by general practitioners causes the unexplained variations, the introduction of capitation-based budgets would gradually move practices with high prescribing costs towards the national average. It is also possible, though, that the unexplained individual variation in prescribing costs is the result of differences in the clinical characteristics or socio-economic conditions of practice populations. If this is the case, capitation-based budgets may lead to unfair distribution of resources. The ASSET age/sex weightings should be used as a guide, not as the ultimate determinant, for an equitable allocation of prescribing resources to regional authorities and general practices.

## Introduction

### Background

The Italian public funding of pharmaceutical prescribing is rapidly evolving from a state centric model to one based upon the equilibrium of central governance of demand and regional funding. The state has exclusive power to define the basic pharmaceutical coverage, which must be uniformly provided across the country, while each Regional Health Authority (ASSR) is responsible for funding the prescribing costs. Equity of access to drug treatment on the basis of clinical need alone remains the central principle of the national healthcare system, raising the issue of an equitable distribution of resources in proportion to the population needs. Since 2004, AIFA, the Agency for Italian Drug Administration, is responsible for the governance of public pharmaceutical prescribing. Besides its regulatory, pricing and reimbursement functions, AIFA is responsible for maintaining the level of public pharmaceutical spending below the threshold of 13% of total public healthcare costs. In case of overspending, AIFA can apply generalised price reductions or modify the level of national pharmaceutical coverage, by delisting entire classes of drugs from the reimbursement list or by limiting the prescription of reimbursed medicines to specified indications. Regional Authorities (ASSR) cannot modify the level of pharmaceutical coverage, but they are entitled to increase local taxes and to apply a prescription fee in order to secure an adequate funding of regional pharmaceutical demand [1].

Many ASSRs are considering introducing capitation based prescribing budgets for their general practices. There are two important factors driving this process: the first is cost containment. It is assumed that budgets will encourage general practitioners to examine their prescribing more critically, resulting in more cost effective and appropriate prescribing. The second factor behind the increasing interest in budgets is the belief that such budgets will help to ensure that resources are allocated more fairly among general practices. The implicit assumption is that, over a number of years, practices will move towards the average and that variation in prescribing costs between practices will be reduced [2].

Weighted capitation based budgets seemed to offer the British National Health Service (NHS) a solution to tackling the dual problem of variations in prescribing costs and increasing drug costs in general practice [3]. Derived by the Prescribing Research Unit (PRU) in 1993, the Age, Sex and Temporary Residents Originated Prescribing Units (ASTRO-PUs) were designed to weight individual practice populations for age, sex and temporary residents. The subsequent introduction of cost-based ASTRO-PUs to allocate prescribing funds and to compare the costs of prescribing between practices was widely criticised [4]–[6].

In particular, Smith argued that the formula did not reflect all patients' related variations in costs, random variations in clinical needs, and differences in clinical practice [7].

Sheldon et al (1994) failed to find any convincing evidence that factors of need other than age and sex were associated with variations in healthcare utilization [8]. The Specific Therapeutic group Age/sex Related Prescribing Units (STAR-PUs), based on British National Formulary (BNF) chapters, were introduced in 1995 as a way of accounting for differences in demography when considering prescribing in different therapeutic areas. These weights were reviewed and revised in 1997 [9]. A study commissioned by the NHS Executive examined the determinants of NHS prescribing expenditures at practice level by relating costs to population needs. The model was based on four variables: permanent sickness, percentage of dependants in no carer households, percentage of students, and percentage of births in the practice list. Together with adjustments made for differences in ASTRO (97)-PUs, the derived robust needs based capitation formula was capable of explaining 62% of variations in prescribing expenditures at practice level [10].

Understanding the determinants of demand for pharmaceuticals is critical for a better assessment of the forces that increase prescribing expenditures. Ageing and technological change play a major role in this context with cohorts living longer that consume increasing amounts of intensive, previously unavailable treatments. More sophisticated econometric models recognised the relevance of clinical determinants to the demand for prescribing, such as morbidity and mortality standardised ratios, chronic illness rates and physicians' prescribing behaviour. Other socio-economic factors, like patients' disposable income, level of education and access to healthcare, also influence the utilisation of pharmaceutical treatments [11].

The primary objective of this study was to make the first step in the modelling of pharmaceutical demand in Italy, by deriving a weighted capitation model to account for demographic differences among general practices. The experimental model was called ASSET (Age/Sex Standardised Estimates of Treatment). Most of the existing models of demographic predictors of prescribing costs have been developed in England, where the National Health Service (NHS) has been adopting capitation based formulae, adjusted for age, sex, morbidity and socio-economic factors for allocating prescribing budgets. Similarly to NHS, the Italian Healthcare System is single payer based, but the development of funding formulae has been delayed by the availability of quality data at individual level. It is becoming increasingly common for local (ASL) and regional (ASSR) Healthcare Authorities to maintain a network of departmental electronic databases, making it possible to integrate all relevant information in the analysis of the trends of pharmaceutical utilization. Most of socio-economic data are still available at aggregate level, raising the need for an accurate age/sex standardisation of prescribing costs among different cohorts. The ASSET model provides a fundamental pre-requisite to a further development of capitation based formulae at regional level.

Prescribing cost data have several characteristics that make them a challenge to analyse. In this paper we discuss the methodological and practical implications for policy makers, healthcare administrators and general practitioners related to the adoption of a weighted capitation formula, trying to answer the following basic questions:

1. What is the formula to use to allocate prescription budgets to general practices equitably?
2. How well does the formula explain the changes in total prescribing costs over time?
3. How well does the formula explain the variability of individual prescription costs in a single year?
4. If the formula were adopted, what would the main implications be for policy makers and general practitioners?

The ASSET model (Age/Sex Standardised Estimates of Treatment) model provides a research-based contribution to these controversial issues in general practice.

## Methods

Patient and cost data were obtained directly from participating local (ASL) and regional (ASSR) healthcare authorities collecting computerised prescription records for a two year period, from January 2004 to December 2005. In particular, the demographic database provided information on subjects' date of birth, sex and healthcare identification number, while prescription data (including patient's name and healthcare identification number, date of issue, name of prescribing physician, brand name of the drug(s) prescribed, generic name, ATC classification, cost and patient's co-payment) was extracted from the territorial pharmaceutical database.

All personal data (name and identification number) were replaced by a univocal numerical code, making both databases anonymous at source in strict compliance with the Italian Privacy Law (Decree 196, 30/06/2003). The study design, observational and retrospective in nature, did not require a previous informed consent from the subjects included (Decree 196/03, art. 110).

The univocal numerical code, attributed to all subjects included in the analysis, made it possible to retrospectively match demographic patient's data with individual prescription costs.

This study was based on data from a 12 month period, as complete patient and cost data from all sources were available from October 2004 to September 2005. No major change in pharmaceutical demand or supply took place during the observation period. The total public expenditure for pharmaceuticals was reported to be €12.4 billion in 2005 and €12.6 in 2004 [12].

The ASSET sample, collected from the ASL of Monza, the ASSR Marche and the ASSR Basilicata, totalled 3,175,691 residents.

Table 1 shows the number of subjects and the percentage in each age/sex group for the sample and for the 2005 population estimates provided by the Italian National Institute of Statistics (ISTAT) [13].

**Table 1:** Distribution of ASSET clusters compared to the distribution of Italian population (ISTAT, 2005)

	ASSET sample size				Italian population			
Age group	Male	Female	Male%	Female%	Male	Female	Male%	Female%
<14	212,014	198,037	6.68%	6.24%	4,242,020	4,013,692	7.26%	6.87%
15–24	158,020	151,022	4.98%	4.76%	3,124,386	2,974,480	5.34%	5.09%
25–34	222,054	218,140	6.99%	6.87%	4,340,899	4,227,651	7.43%	7.23%
35–44	261,120	254,452	8.22%	8.01%	4,728,844	4,678,965	8.09%	8.00%
45–54	212,537	216,539	6.69%	6.82%	3,816,508	3,903,129	6.53%	6.68%
55–64	185,003	194,651	5.83%	6.13%	3,406,977	3,625,483	5.83%	6.20%
65–74	167,297	191,304	5.27%	6.02%	2,792,032	3,322,000	4.78%	5.68%
>75	127,172	206,329	4.00%	6.50%	1,925,138	3,340,171	3.29%	5.71%
Total	1,545,217	1,630,474	48.66%	51.34%	28,376,804	30,085,571	48.54%	51.46%

Using the 2005 ISTAT figures as a base (see Table 1), the difference between the sample and the expected distribution of population could be calculated using a chi-square goodness of fit test. The statistic was 17,064 with 15 degrees of freedom, which was highly significant (p = 0.000). The null hypothesis that the ASSET sample and the Italian population had a similar age/sex distribution could be rejected. The most significant differences were in both tails of the distribution: the ASSET model had a larger number of subjects aged <14 and >75 compared to the distribution of population. Although the differences in Table 1 are highly statistically significant, this is largely a function of the number of patients in each cell of the table. The absolute differences between the ASSET population and the Italian population are small: similarly to STAR-PUs, since the ARTE weightings were derived from the cost and number of items per patient, rather than from the total costs and number of items, this should not necessarily affect the quality of the measures [14].

Individual cost data referred to the pharmaceutical spending over the 12-month period between October 2004 and September 2005. Pharmaceutical spending was defined as the total individual cost for reimbursed drugs only (class A), dispensed by retail pharmacies (not including hospital consumption), at actual prices including co-payment. During the observation period, co-payment was limited to a fixed prescription fee amounting to €1/€2 depending on the number of items per prescription. In 2005, total co-payments amounted to 3.8% of total prescribing costs [12]. Excluded from the analysis were special drugs dispensed from hospital pharmacies and out-of-pocket expenses for non-reimbursed drugs (class C).

The average prescribing costs by age group were calculated simply by dividing the total pharmaceutical cost per age group by the total number of subjects in the same age bracket. Note that the number of subjects was the number registered in the demographic database and not the number of persons receiving prescriptions. Weights were obtained by dividing each average prescribing cost by the total average cost (€195.6). Let *i* be the age/sex group,
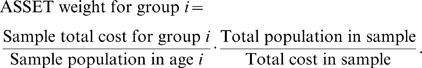



The ASSET weighting method was different from the one adopted by the British STAR/ASTRO-PUs, where each average cost by age group was divided by the average cost of the 0–4 age group. The choice of total average cost as a weighting constant was recommended by those Healthcare Administrators who presently do not have access to computerised patient records. While they cannot derive the average cost by individual age groups, they can still calculate the total average prescribing cost (total pharmaceutical expenditure divided by total assisted population). The constant known, they can derive a pro-forma age/sex weighted budget for each individual practice based on prior year prescribing costs.

The cost data reported in Table 2 allow to easily calculating the weights consistently to the STAR/ASTRO-PUs formula. A strong caveat to any direct comparison between the two sets of weightings cannot be overemphasized, as they reflect different reimbursement and prescribing policies, drug prices, prescribing behaviours and observation periods.

**Table 2:** ASSET's mean values by age group and standardised weights for overall prescribing.

	Mean cost (Euro)		Standardised weights	
Age group	Males	Females	Males	Females
<14	41.37	35.72	0.21	0.18
* 0–4*	*36.11*	*31.59*	*0.18*	*0.16*
* 5–14*	*43.66*	*37.53*	*0.22*	*0.19*
15–24	44.93	40.94	0.23	0.21
25–34	52.75	62.75	0.27	0.32
35–44	80.89	90.52	0.41	0.46
45–54	146.20	149.62	0.75	0.76
55–64	300.88	277.40	1.54	1.42
65–74	505.77	431.13	2.59	2.20
>75	652.75	481.20	3.34	2.46
**Total**	**196.13**	**195.12**	**1.00**	**1.00**

The ASSET model grouped all patients into16 age groups, differently from the 18 weightings used by the STAR/ASTRO-PUs. The 0–4 and 5–14 age groups were aggregated into a single 0–14 cluster, since in Italy children under 15 years are mandatory seen by Paediatricians. Table 2 reports the breakdown of the 0–14 age cluster into 0–4 and 5–14 separate groups (in italic), but the relative weightings were not utilised in the prescribing costs analysis.

## Results

### The ASSET model

To derive a relationship between demography and prescribing costs, individual cost data were collected for 3,175,691 subjects living in three different regions of Italy (Lombardy, situated in the north, Marche in the centre and Basilicata in the south). The observation period was 12 months (Oct 2004–Sep 2005).

The standardized weights given in Table 2 were obtained by dividing the mean cost of each age group by the total mean cost (€195.6).

The distribution of both male and female subjects included in the ASSET sample was non-normal, with a mode at 0 and a heavy right tail. The mean annual prescribing cost per individual was similar for males (€196.13) and females (€195.12), but the distribution of medicine utilization by age showed significant differences (above 10%).

Differently from what observed in England, in Italy prescribing costs among young children (0–4 years) were lower than costs among older children (5–14). This was not surprising: in the Italian Healthcare system, expensive neo-natal treatments are directly dispensed by hospital and local ASL and this specific drug distribution was not captured by the ASSET cost data.

Compared to males' mean costs of treatment, female drug utilization was lower in the pubescent and teens age (first two brackets), higher in the adult life (third and forth bracket), fairly similar in the fifties (fifth bracket), to increase at a significant lower rate than males in the senior years (from 55 to death). Taking into account that neither contraceptives (not reimbursed) nor expensive fertility drugs (delivered in hospital) were included in the prescription costs analysed, the reasons for these time-lagged discrepancies remained unexplained and they would be worth further investigation.

**Figure 1:**
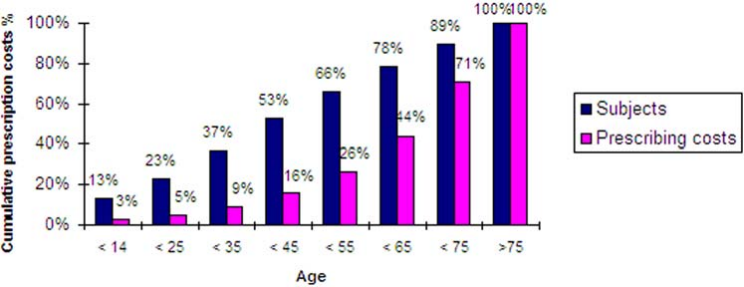
ASSET sample cumulative distribution of prescribing costs by age.

After 65 years of age, the mean prescribing costs for males were significantly higher than females. On average, a 75 year old subject would cost 12 times a 25–34 years old one if male, and 8 times if female. Figure 1 shows that subjects over 65 years of age (22% of total population) accounted for 56% of total prescribing costs.

To evaluate the error embedded in the age/sex distribution of the sample size, the ASSET weightings were used to estimate 2005 total prescribing costs. The cost estimate was calculated by adding the costs by age/sex group obtained by multiplying the number of Italian residents in each group by the relative weight and subsequently by the actual mean pharmaceutical public spending per resident published by AIFA (€211.5). The estimate of total costs was then compared to the 2005 total prescribing costs, reported by AIFA as €12.63 billion, including co-payment. The ASSET age/sex model estimated 94% of total 2005 Italian prescribing costs.

The main factor to cause the variance between the ASSET weightings based estimate and the actual total Italian prescribing cost was the cost of the non-assignable items, not included in the calculation of weights (4.23% of total ASSET costs). Most of the non-assignable items were prescribed to patients whose identity was not included in the demographic databases, possibly either because they were non-resident or because they just moved in from a different healthcare district. The demographic databases are aligned with census data at a given frequency; therefore a time lag effect is always to be taken into consideration when measuring the accuracy of cost data analysis for large cohorts. Less than 1% of the total analysed prescription data were non-assignable due to errors in compilation or missing data.

### Testing the explanatory power of the ASSET model

The ASSET weightings were used to retrospectively explain the evolution of total Italian prescribing costs of reimbursed drugs (class A) between 2000 to 2005.This relatively short time frame was chosen because age/sex weightings reflect clinical needs and prescribing patterns that may change over a longer period of time, due to the availability of improved diagnostic tools or innovative therapeutic options [15].

Table 3 shows the 2000–2005 Italian resident population weighted using the ASSET's weights to highlight inequalities in the age/sex distribution (ageing, adult immigration) that could have had an impact on prescribing demand, measured as total public spending on pharmaceuticals (including co-payment).

**Table 3:** 2000–2005 annual Italian resident population (ISTAT), resident population weighted using the ASSET's weights and total pharmaceutical spending on reimbursed drugs, including co-payment (AIFA).

Year	Italian Population	Weighted Population	Total cost (Mill euro)
**2005**	58,462,375	55,018,918	12,363
**2004**	57,888,245	54,129,403	12,580
**2003**	57,321,070	53,259,666	11,737
**2002**	56,993,742	52,403,499	12,060
**2001**	56,960,692	51,860,378	11,621
**2000**	56,923,524	51,381,248	9,625

The explanatory power of the ASSET model was tested by regressing the five year trend of total public pharmaceutical expenditures and the ASSET-weighted population residuals. The weighted Italian population was obtained by adding the number of residents by each age/sex group multiplied by the relative ASSET weight:
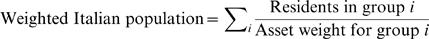


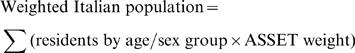



Due to the limited number of regression points, simply rejecting the null hypothesis was not very informative, since the very low power of the test would give a high probability of type II errors. Although we used a 10% significance level, instead of the classical 5%, the significance tests must be read with caution, keeping in mind that the degrees of freedom were too few to carry out a meaningful inference.

The weighted population's p-value (0.1028) was on the boundary of the rejection region, but the F test (11.19) for the overall regression was significant. The R-square was 0.88, but with 3 degrees of freedom was not surprising. The negative coefficient of the weighted population was also expected, since it was certainly collinear with the time trend, and its action in this model was to correct for the age structure of the population.

Taking into account the incremental nature of prescribing costs, we tested the impact of a time trend on the regression outcomes. Adding a linear variable (years) to the regression did not change the R-square (0.88) and the significance (F test: 11.18) of the model.

The interpretation of this regression model is that the total public prescribing costs are growing faster than the ratio underlying the ASSET model (constant spending for age/sex groups) would predict.

Besides confirming the relevance of age and sex distribution as predictors of prescribing demand, this outcome was particularly interesting, taken into account the continuous effort operated by AIFA to centrally maintain the level of pharmaceutical spending below the threshold of 13% of total healthcare spending. A number of actions were taken during those years to reduce prescribing costs, such as:

1. De-listings of drugs from reimbursed status (Class A);
2. Generalised price cuts;
3. Prescribing limitations;
4. Direct distribution of innovative, expensive treatments;
5. Generic substitution.

The power of the ASSET model to explain individual cost variability was determined by fitting a linear regression analysis using the log transformed actual individual costs reported by 50,000 subjects randomly drawn from the sample, as the dependent variable and the log transformed mean costs by age group as the independent variable. Cost data are often transformed to the log scale, which shortens the long right tail, lessens heteroskedasticity, and decreases the influence of outliers [16].

An equal probability sample of 50,000 patients was drawn from the ASSET database using the randomization procedure of Oracle Dynamic Sampling. The random sample included subjects showing no prescription in the year (zero cost).

Due to the number of zero prescription costs in the sample, it was assumed that the conditional distribution of the prescription costs was a mixture of two distributions: a distribution that puts all its probability mass in the point 0 and a log-normal distribution. The maximisation of the likelihood could be split in two steps: first a binary model to estimate the probability that a patient would receive a prescription in the 12 month observation period (Y>0), and then a log-normal regression model for the positive Y values could be estimated.

The likelihood function of the model was: 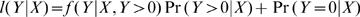
(1)


For the binary model, the McFadden R-square was 0.13. All model's parameters were highly significant. Standard errors were based on a robust Huber/White covariance matrix. The sign of the regressor was positive as expected.

For the log-normal regression, the standard errors have been calculated using White's heteroskedasticity-consistent matrix, since White's test indicated possible heteroskedasticity. The R-square (limited to persons with non-zero prescriptions) was 0.25. The Jarque-Bera test for normality (as well as other common normality tests) rejected the normality hypothesis, but this is not uncommon with so many observations in sample.

The expected value of the prescription cost was given by: 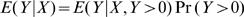
(2)


Few extreme underestimations dominate the variance of the prediction error [*Y*−*E*(*Y*|*X*)]: the maximum error was circa €27,912, that was almost 20 times larger than the absolute value of the minimum error (€1,483).

In order to check if alternative transformations of the two variables could yield better results we estimated the double Box-Cox regression
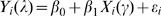
(3)with *Y_i_*(*λ*) and *X_i_*(*γ*) indicating Box-Cox transforms with parameters *λ* and *γ* , by Gaussian maximum likelihood. (Notice that since we are supposing the conditional normality of a transform of our data, we have to modify the likelihood according to the Jacobian of this transformation. The correct form of the log-likelihood may be found, for example, on page 500 of Green W. (1993) *Econometric Analysis*, 5^th^ edition, Prentice Hall.) Since we are supposing the conditional normality of a transformation of our data, we have to modify the likelihood according to the Jacobian form of this transformation [17]. The residual distribution and the R-square (0.26) of this model are only slightly better then the previous one, even though formal tests for *λ* = 0 and *λ* = 1 reject the null at any usual significance level. By looking at the estimated Box-Cox transform parameters, the logarithm seems a sensible choice for the dependent, while a square root seems reasonable for the regressor. Although the results are significant the difference between the two estimated models seems not so relevant form a practical point of view.

Beyond the technicalities of the analysis, the important finding was that the ASSET model could not explain large variations in individual prescribing costs. This was not a surprising outcome: in regression analyses of healthcare utilization data, the R-square values were usually on the order of <20%. Newhouse used theoretical and empirical arguments to estimate that the maximum possible R-square was about 48% for outpatient costs [18].

As discussed earlier, to increase the explanatory power of capitation based models, additional determinants of prescribing demand should have been considered, such as morbidity and mortality ratios, chronic illness rates, deprivation and access to healthcare, together with other relevant socioeconomic determinants, like disposable income and level of education. The ASSET model was a first step in developing a more rational approach of allocating prescribing funds in Italy.

## Discussion

### Implications for Italian healthcare policy makers

Policy makers not only need to know the determinants of public prescribing expenditures, but they should also have the possibility to estimate the impact of those trends having a significant impact on pharmaceutical demand.

The ageing of population is a known fact. According to ISTAT data, in the last twenty years, life expectancy at birth increased by 6 years for males (76.9 years) and by 5 years for females (82.9 years). The ASSET model confirms the strong, quasi-exponential relationship between age and pharmaceutical utilization, allowing policy makers to quantify the impact of ageing population in terms of resources needed to satisfy the incremental therapeutic needs.

As an example, the intermediate scenario of the latest population projections foresees in 2026 a marginal decrease in the total number of Italian residents (57.5 million), down by 1.6% compared to the current 58.5 million inhabitants [19]. All else equal (prices, therapeutic alternatives, and public coverage of prescription costs), we could assume that prescription costs should remain relatively stable over the next two decades. The ASSET model helps policy makers and demographic statisticians to actually demonstrate the opposite.

Multiplying the ASSET weights by the expected number of residents, we obtain an age/sex-standardised population that reflects the relative cost of pharmaceutical utilization. A 0–14 year old male, on average, accounts for just one fifth of the mean annual prescribing cost, while a 75 year old male uses 3.3 times as many medicines as the average. Population data standardised with the ASSET's weights represent a close proxy of pharmaceutical spending. In table 4 we derive the weighted Italian population estimated for 2026 using the ASSET weights. The weighted population is expected to grow from 55 million in 2005 (see Table 3) to 65.8 million in 2026. All else equal, the pharmaceutical spending in 2026 is likely to be almost 20% higher than in 2005 as a result of the ageing population.

**Table 4:** 2026 Italian resident population projected by ISTAT (intermediate scenario) weighted using the ASSET's weights.

Age groups	Estimated Italian population in 2026		ASSET weights		2026 weighted population	
	Male	Female	Males	Females	Males	Females
<14	3,524,980	3,321,139	0.21	0.18	745,463	606,469
15–24	2,909,978	2,759,286	0.23	0.21	668,435	577,535
25–34	3,083,146	2,958,039	0.27	0.32	831,462	948,853
35–44	3,376,580	3,239,167	0.41	0.46	1,396,231	1,498,881
45–54	4,237,566	4,133,935	0.75	0.76	3,167,131	3,162,039
55–64	4,545,376	4,587,154	1.54	1.42	6,991,435	6,505,063
65–74	3,311,146	3,679,641	2.59	2.20	8,561,232	8,110,045
>75	3,048,694	4,805,801	3.34	2.46	10,173,432	11,822,110
**Total**	**28,037,466**	**29,484,162**			**32,534,821**	**33,230,996**

The ageing process shows wide regional variability. Regions with the highest percentage of residents over 65 years old are located in the North and Centre of Italy: Liguria (26.2%), Umbria (23.1%), Toscana (22.9%), Friuli and Piedmont (21.8%). The Southern regions show the lowest percentage of elderly residents: Campania (14.7%), Puglia (16.5%) and Sardinia (16.6%).

Policy makers must allocate adequate resources to regions to fund prescribing costs based on clinical needs rather than population density. A simple capitation formula would ignore differences in demographic distribution among regions, inevitably under-funding those areas with the highest concentration of elderly population. For example, consider two Italian regions, Liguria (North East) and Sardinia (island), that have a similar population of 1,6 million and 1,7 million resident respectively, but a ten percent points difference in the elderly population (22.9% vs. 16.6% residents over 65 years old).

A straight capitation formula would allocate to Liguria a prescription budget 3.6% lower than the one allocated to Sardinia. Comparing the number of residents weighted by age and sex (1.8 million in Liguria vs. 1.5 million in Sardinia), we realise that Liguria actually needs 18% more prescribing funds than Sardinia to cover the therapeutic needs of its older population (Table 5).

**Table 5:** Comparison of 2005 population of two Italian regions (Liguria and Sardinia) weighted using the ASSET's weights.

Age groups	Liguria's weighted residents		Sardinia's weighted residents	
	Male	Female	Male	Female
<14	18,916	15,476	23,618	19,063
15–24	14,219	12,289	22,924	19,761
25–34	26,811	30,923	35,436	40,688
35–44	51,603	57,163	55,301	62,074
45–54	77,387	81,771	86,520	89,428
55–64	160,759	164,405	148,695	143,871
65–74	248,963	261,830	184,815	186,219
>75	245,121	329,431	163,786	191,373
**Total**	**843,779**	**953,288**	**721,094**	**752,476**

The ASSET model is a useful tool to support the process of long term healthcare policy planning as well as the equitable allocation of annual prescribing resources to regional authorities.

### Implications for regional healthcare administrators

Regional healthcare authorities (ASSR) could use a similar mechanism to equitably allocate prescribing cost guidelines to general practices on the basis of population need.

Should two practices with a similar number of patients each receive the same level of prescribing funds? Not necessarily. The demographic differences in general practices ought to be recognised by the formula used to allocate the pharmaceutical budget.

Let's compare two general practices, A&B. Both practices have the maximum number of patients (1,500) allowed by the Italian Healthcare System and the proportion of male and female patients is approximately the same for both practices (47% male vs. 53% female). The age distribution, though, differs significantly between the two practices: 935 patients (62% of total) in practice A are older than 65, compared to 642 patients (43% of total) in practice B. Table 6 shows that while a simple per capita distribution of resources would allocate to both practices an equal prescribing budget , the ASSET model reflects both the size of the practice list (1,500 patients each) and its age and sex structure in the budget allocation , granting to practice A (3,020 weighted patients) a prescribing budget 13% higher than practice B (2,621 weighted patients).

**Table 6:** Hypothetical prescribing budget allocation using the ASSET weights to two practices (A&B) with equal number of patients, similar male/female ratio, but significantly different age distribution.

Age groups	Actual population				Weighted population			
	Practice A		Practice B		Practice A		Practice B	
	Male	Female	Male	Female	Male	Female	Male	Female
<14								
15–24	12	10	55	50	3	2	13	11
25–34	22	26	48	60	6	8	13	16
35–44	43	48	63	73	18	22	26	30
45–54	65	68	75	128	48	52	56	96
55–64	134	137	144	162	206	195	221	249
65–74	208	232	168	169	537	511	434	437
>75	220	275	155	150	734	676	517	501
**Total**	**704**	**796**	**708**	**792**	**1,553**	**1,467**	**1,281**	**1,340**

### Implications for general practitioners

If health authorities are considering the introduction of capitation based budgets then general practitioners will need to prepare for this. They should familiarise themselves with the basic methods of cost analysis in order to understand the factors that can increase the demand for medicines and to be able to discuss the wide variations in prescription costs among patients.

Demographic adjusted healthcare cost models, such as ASSET, tend to lose their explanatory power when the subset of population examined gets smaller. As discussed in the previous section, while the ASSET model is able to explain 60% of the five-year pharmaceutical cost trend of Italy, a country of 58 million inhabitants, its ability to explain individual utilization differences goes down to approximately 30% for a sample of 3.1 million subjects. When the unit of analysis is a single practice, idiosyncratic prescribing causes may overshadow differences in clinical characteristics of practice population, such as the incidence of diabetes, asthma or ischaemic heart disease.

A weighted capitation base formula would then classify practices only as low cost or high cost prescribers, telling nothing about the quality of prescribing, an essential determinant of demand. This information can only come from a detailed analysis of practice's prescribing data combined with information directly collected from each practice.

The implementation of the ASSET capitation formula would provide an effective benchmark to compare prescribing costs standardised by age and sex differences in the practice list, but is just a starting point in the process of optimization of prescribing resources It should help practitioners to reflect upon specific determinants of demand for medicines in their practice, such as the transfer of care from hospitals to general practice or a high prevalence of chronic diseases, to identify areas in which costs could be saved through a more rational prescribing.

### Conclusions

The ASSET age/sex standardisation model, therefore, proved to be a useful, but not an exhaustive tool to equitably align the distribution of resources among regions according to their relative ageing rate.

The ASSET weightings were able to explain only about 25% of the variation in prescribing costs among individuals: the causes of the remaining 75% variation in prescribing costs remained unknown. The magnitude of individual variance was extremely significant: the individual costs value in the ASSET sample ranged between 0 and >40,000 euros. The ASSET sample included the registered persons who did not receive any prescription in the same time period: 808,464 subjects (26% of the total sample) did not receive a prescription, of whom 488,120 males (32% of total males) and 320,344 females (20% of total females).

From a different perspective, the ranking by total pharmaceutical annual cost of the 50,000 individuals included in the randomly drawn sample utilised to test the ASSET model, showed that the first decile of highest spending subjects was associated with 51.4% of total pharmaceutical spending. The derivation of a robust model capable of identifying the drivers of individual variances should be the objective of further research. If mainly idiosyncratic prescribing by general practitioners causes the unexplained variations, the introduction of capitation-based budgets would gradually move practices with high prescribing costs towards the national average. It is also possible, though, that the unexplained individual variation in prescribing costs is the result of differences in the clinical characteristics of practice populations or because some general practices are better at early diagnoses, treatment and compliance of patients suffering from chronic conditions. If this is the case, capitation based budgets may lead to unfair distribution of resources [20].

The ASSET age/sex weightings should be used as a guide, not as the ultimate determinant, for an equitable allocation of prescribing resources to regional authorities and general practices.

## References

[1] Giarda DP, Petretto A, Pisauro G, Lorenzini S, Vignocchi C (2005). Elementi per una politica di governo della spesa pubblica. Paper presented at the conference “Oltre il Declino”, Fondazione Rodolfo Debenedetti, Rome, February 3.. http://www.frdb.org.

[2] Majeed A, Head S (1998). Capitation based prescribing budgets will not work.. BMJ.

[3] Roberts SJ, Harris CM (1993). Age, sex, temporary resident originated prescribing units (ASTRO-PUs): new weightings for analysing prescribing of general practices in England.. BMJ.

[4] Coulter A (1994). Capitation funding may over fund practices in better off areas.. BMJ.

[5] Baker Y (1998). Use of capitation formulas for primary care groups could result in chaos.. BMJ.

[6] Carr-Hill R, Roberts D (1999). Population figures for capitation formulas need to be designed differently.. BMJ.

[7] Smith P, Sheldon TA, Carr-Hill RA, Martin S, Peacock Hardman G (1994). Allocating resources to health authorities: results and policy implications of small area analysis of use of inpatient services.. BMJ.

[8] Sheldon TA, Smith P, Borwitz M, Martin S, Carr-Hill R (1994). Attempt at deriving a formula for setting general practitioner fund holding budgets.. BMJ.

[9] Lloyd DC, Harris CM, Roberts DJ (1995). Specific therapeutic age-sex related prescribing units (STAR-PUs): weightings for analysing general practices' prescribing in England.. BMJ.

[10] Rice N, Dixon P, Lloyd DCEF, Roberts D (2000). Derivation of a need based capitation formula for allocating prescribing budgets to health authorities and primary care groups in England.. BMJ.

[11] Majeed A, Malcolm L (1999). Unified budgets for primary care groups.. BMJ..

[12] Agenzia Italiana del Farmaco AIFA (2005). L'uso dei farmaci in Italia. OSMED reports 2000–2005.. http://www.agenziafarmaco.it/aifa/servlet/section.ktml?target = &area_tematica = ATTIVITA_EDITORIALE&section_code = AIFA_PUB_RAP_OSMED&cache_session = true.

[13] Istituto Nazionale di Statistica (ISTAT) Demographic statistics analysis on the Italian population 2004/2005.. http://demo.istat.it/index.html.

[14] NHS ASTRO-PUs and Chapter STAR-PUs: 2001 revision paper.. http://www.ic.nhs.uk/psu/measure/presmeas/copy_of_atropus.

[15] Smith PC (1999). Setting budgets for general practice in the new NHS.. BMJ.

[16] Diehr P, Yana D, Ash A, Hornbook M, Lin DY (1999). Methods for analyzing healthcare utilization and costs.. Annual Review of Public Health.

[17] Green W (1993). Econometric analysis.. Prentice Hall, 5^th^ edition.

[18] Newhouse JP, Manning WG, Keeler EB, Sloss EM (1989). Adjusting capitation rates using objective health measures and prior utilization.. Healthcare Finance Review.

[19] Istituto Nazionale di Statistica (ISTAT) (2051). Demographic projections of the Italian population by age, sex and region: 2001/2051.. http://demo.istat.it/prev/index.html.

[20] Whynes DK, Baines DL, Tolle KH (1996). Explaining variations in general practice prescribing costs per ASTRO-PU (age, sex and temporary resident originated prescribing unit),. BMJ.

